# Diagnosis, treatment, and response assessment in solitary plasmacytoma: updated recommendations from a European Expert Panel

**DOI:** 10.1186/s13045-017-0549-1

**Published:** 2018-01-16

**Authors:** J. Caers, B. Paiva, E. Zamagni, X. Leleu, J. Bladé, S. Y. Kristinsson, C. Touzeau, N. Abildgaard, E. Terpos, R. Heusschen, E. Ocio, M. Delforge, O. Sezer, M. Beksac, H. Ludwig, G. Merlini, P. Moreau, S. Zweegman, M. Engelhardt, L. Rosiñol

**Affiliations:** 1 Department of Hematology, CHU de Liège, Liège, Belgium; 2 Clinica Universidad de Navarra, Centro de Investigacion Medica Aplicadas (CIMA); Instituto de Investigación Sanitaria de Navarra (IDISNA), CIBERONC, Pamplona, Spain; 3 Seràgnoli Institute of Hematology, Bologna University School of Medicine, Bologna, Italy; 4 Hopital La Miletrie, University Hospital of Poitiers, Poitiers, France; 5 Department of Hematology, Hospital Clínic de Barcelona, IDIBAPS, Barcelona, Spain; 6 Department of Hematology, Landspitali National University Hospital, Reykjavik, Iceland; 7 Department of Hematology, University Hospital Hôtel-Dieu, Nantes, France; 8 Department of Hematology, Odense University Hospital, Odense, Denmark; 9 Department of Clinical Therapeutics, School of Medicine, National and Kapodistrian University of Athens, Athens, Greece; 10 Instituto de Investigacion Biomedica de Salamanca, Centro de Investigación del Cancer, Hospital Universitario de Salamanca, Salamanca, Spain; 11 Department of Hematology, University Hospital Leuven, Leuven, Belgium; 12 Department of Haematology, Oncology, and Bone Marrow Transplantation, Universitaetsklinikum Eppendorf, Hamburg, Germany; 13 Department of Hematology, University of Ankara, Ankara, Turkey; 14 Department of Medicine I, Wilhelminen Hospital, Vienna, Austria; 15 Department of Molecular Medicine, Amyloidosis Research and Treatment Center, Foundation ‘Istituto di Ricovero e Cura a Carattere Scientifico (IRCCS) Policlinico San Matteo’, University of Pavia, Pavia, Italy; 16 Department of Hematology, VU University Medical Center, Amsterdam, the Netherlands; 17 Department of Hematology and Oncology, University of Freiburg Medical Center, Freiburg, Germany

**Keywords:** Solitary plasmacytoma, Extramedullary plasmacytoma, Myeloma, Plasma cell dyscrasia, PET/CT, MRI, Radiotherapy

## Abstract

Solitary plasmacytoma is an infrequent form of plasma cell dyscrasia that presents as a single mass of monoclonal plasma cells, located either extramedullary or intraosseous. In some patients, a bone marrow aspiration can detect a low monoclonal plasma cell infiltration which indicates a high risk of early progression to an overt myeloma disease. Before treatment initiation, whole body positron emission tomography–computed tomography or magnetic resonance imaging should be performed to exclude the presence of additional malignant lesions. For decades, treatment has been based on high-dose radiation, but studies exploring the potential benefit of systemic therapies for high-risk patients are urgently needed. In this review, a panel of expert European hematologists updates the recommendations on the diagnosis and management of patients with solitary plasmacytoma.

**Electronic supplementary material:**

The online version of this article (10.1186/s13045-017-0549-1) contains supplementary material, which is available to authorized users.

## Background

Plasma cell (PC) neoplasms can present in different clinical forms. Multiple myeloma (MM) is generally located in the bone marrow (BM) and associated with a wide spectrum of clinical, laboratory, and radiological findings [1]. Conversely, solitary plasmacytoma (SP) is characterized by a single mass of clonal plasma cells, with no or minimal BM plasmacytosis and with no other symptoms than those derived from the primary lesion. It can present either as extramedullary (extraosseous) plasmacytoma (EMP), i.e., in soft tissues, or as solitary bone plasmacytoma (SBP). SP is a rare condition with a cumulative incidence of 0.15/100.000 [2]. Liebross et al. reported that out of 1354 patients treated for plasma cell neoplasms at the MD Anderson Cancer Center between 1963 and 1996, 1272 patients (94%) had MM, 60 patients (4%) had SBP, and 22 patients (2%) had EMP [3]. A recent Swedish population study showed a similar distribution of patients, with a global incidence of 0.191/100.000 for male and 0.090/100.000 for female patients [4]. SBP comprises 70% of all SP cases and occurs primarily in red marrow-containing bones such as vertebrae, femurs, pelvis, and ribs. EMP can involve any site or organ, with the most frequent being the head and neck region (sinuses, naso- and oropharynx), gastrointestinal tract, and lungs [3, 5]. Patients presenting with SBP, especially those cases with minimal BM plasmacytosis, have a higher risk of developing symptomatic MM: approximately 50% of patients with SBP and 30% of patients with EMP develop MM within 10 years after the initial diagnosis [6]. Here, we provide a consensus statement on the diagnostic criteria, prognostic factors, treatment, and response criteria for SP. Readers should be aware that the management of patients with localized or solitary plasmacytomas is different from patients with soft-tissue plasmacytomas in the context of overt multiple myeloma.

## Methodology

These guidelines were developed by a working group of clinical hematologists with expertise in MM. Literature review was performed up to September 2017 and included published clinical studies, meta-analyses and reviews (Medline), and abstracts from the American Society of Hematology (ASH) and European Hematology Association annual meetings (keywords: plasmacytoma, solitary, myeloma, PET/CT, MRI, radiotherapy, diagnosis, and treatment). Key recommendations were developed based on randomized, controlled clinical trial evidence. If data were insufficient, expert consensus was used to suggest recommendations. The drafted recommendations were circulated among the working group members who all provided comments. In addition, the recommendations were discussed by working group members at the International Myeloma Working Group (IMWG), European Myeloma Network (EMN), and ASH meetings. After three rounds of revisions, all working group members reviewed and validated the final recommendations which were assigned using the GRADE criteria, which incorporate recommendation strength and quality of evidence (Additional file 1: Table S1).

## Definition and diagnostic criteria

The advent of more sensitive techniques to assess BM plasmacytosis has recently led to an updated definition of SP by the IMWG [1]. SPB is defined by the presence of a single lytic lesion due to monoclonal PC infiltration, with or without soft-tissue extension, and EMP consists of a soft-tissue mass that is not in contact with bone [7]. Accordingly, conventional morphology or immunohistochemistry typically show no BM plasmacytosis, but minimal BM infiltration by clonal plasma cells (PCs < 10%) was still considered consistent with a SP diagnosis, provided that no other lesions are observed (Table 1).

**Table 1:** Diagnostic criteria for solitary plasmacytoma and overlapping disorders

		Plasmacytoma	Serum monoclonal protein	Bone marrow cytology	End organ damage	Radiological work-up	Risk of progression
Solitary plasmacytoma	Solitary plasmacytoma	Present	Not required	Negative		No other lesions	10% will progress to MM within 3 years
	Solitary plasmacytoma with minimal bone marrow involvement	Present	Not required	Monoclonal PC infiltration < 10%	Absent	No other lesions	60% with SBP or 20% with EMP will progress to MM within 3 years
Multiple myeloma	Macrofocal myeloma	Present	Not required	Monoclonal PC infiltration < 10%	Possible	Multiple lesions	
	Multiple myeloma	Not required	Present	Monoclonal PC infiltration ≥ 10%	Present	Other lesions could be present	

Abbreviations: *MM* multiple myeloma, *BM* bone marrow, *PC* plasma cell, *EMP* extrameddulary plasmacytoma

SP diagnosis is currently based on a tissue biopsy and histological and immunohistochemical confirmation of the presence of a homogenous infiltrate of monoclonal plasma cells, which typically express CD138 and/or CD38. Monoclonality needs to be proven by kappa/lambda light chain restriction or by a PCR-based approach. Cytogenetic analysis of the SP generally identifies the abnormalities that are frequently encountered in MM. Interphase fluorescence in situ hybridization (FISH) was realized in 2 small studies on EMP and identified a high incidence of 13q losses (ranging from 33 to 40%), IGH rearrangements (in about 37 to 53%), and hyperdiploidy in 54% of the cases [8, 9]. However, no prognostic correlation could be found between chromosomal aberrations and clinical features or disease progression [8].

Two independent studies detected low levels of clonal PCs in the BM by using more sensitive methods (i.e., flow cytometry) [10, 11]. Hill et al. demonstrated in 50 patients with SBP that occult BM disease, defined as a discrete population of phenotypically aberrant PCs, is present at diagnosis in 68% of patients. Importantly, the presence of such aberrant cells had prognostic significance, since progression to symptomatic MM or a new plasmacytoma outside the irradiation field was documented in 72% (26/34) of these patients with occult disease vs. 12.5% (2/16) in patients without and the median time to progression was 26 months vs. not reached. Furthermore, Paiva et al. reported that 17 of 35 (49%) of patients with SBP had aberrant BMPCs [11]. Of interest, 71% of patients with positive flow cytometry evolved to MM vs. only 8% of those with negative flow. This suggests that flow cytometry may be helpful in the distinction of the true SBP (negative flow cytometry) with a very low rate of evolution to MM from those with high risk of progression to myeloma (positive flow cytometry). Both studies confirm that SP patients with minimal BM plasmacytosis have an increased risk of progression to MM compared to patients without BM involvement and close attention should be given to the former group during routine follow-up.

Finally, there is a small but well-recognized group of patients characterized by multiple lytic bone lesions and low BM plasmacytosis, the so-called macrofocal form of MM [12]. These patients are generally younger than the overall myeloma population and have a better prognosis.

### Bone marrow assessment

A unilateral BM aspiration and trephine biopsy is recommended for all patients with suspected SP. In order to exclude > 10% of monoclonal PCs in the BM, a BM aspiration with immunophenotyping to define the proportion of monoclonal cells by kappa/lambda labeling should be performed. When the possibility for immunophenotyping is missing, a BM biopsy is recommended with immunohistochemistry to detect monoclonal PCs. A biopsy might reveal more monoclonal cells because of a sampling error with aspiration. The higher PC count of either aspiration or biopsy should be considered in cases of discrepancy between both techniques.

Noteworthy, the malignant phenotype of clonal PCs among patients with SP resembles that of cases with MM; accordingly, we recommend that laboratories use the same immunophenotypic strategy used in MM such as that established by the EuroFlow consortium for the assessment of minimal residual disease (MRD), which has a median limit of tumor cell detection of 2 × 10^−6^, and is virtually applicable to all patients with PC dyscrasias [13]. BM plasmacytosis > 10% constitutes a definitive MM diagnosis.

**Recommendations:** *All patients with suspected SP should receive a BM aspiration and a BM biopsy to evaluate PC morphology and the degree of total PC infiltration (Grade 1A). Given the diagnostic and prognostic importance, the degree of clonal PC infiltration should be determined by flow cytometry or by kappa/lambda labeling on the BM aspirate or by immunohistochemistry on a BM biopsy (Grade 1B). When a monoclonal PC infiltration is present at baseline, the BM aspiration and biopsy should be repeated when a progression to MM is suspected.*

### Imaging

Detection and localization of SP depends on imaging studies. In addition, since SP is defined by the absence of other bone or extramedullary lesions, imaging is required to exclude a MM diagnosis.

#### Conventional radiography of the skeleton and computed tomography

Conventional radiography of the skeleton (skeletal survey) has been recommended for the initial assessment of bone lesions for decades. SBP preferentially replaces the trabecular bone, while the cortical bone is partly conserved or even sclerotic [14]. In two thirds of the cases, the radiographic appearance is characteristic with a mixed, predominantly lytic pattern. Less commonly, SBP has a multicystic appearance. Similar to in MM patients, skeletal surveys are not sensitive enough to detect early lytic bone lesions which are only visible when more than 30% of cortical bone is destructed [15, 16]. Moreover, conventional X-ray imaging does not reveal EMP located in soft tissues. This underlines the need for other imaging techniques for the evaluation of skeletal and extramedullary lesions, i.e., computed tomography (CT), magnetic resonance imaging (MRI), and positron emission tomography (PET)/CT.

In the context of EMP, CT may be helpful for an adequate loco-regional staging as regional lymph node recurrences occur in 7% of EMP cases. CT can also be used to identify spinal cord and/or nerve root compression when MRI is unavailable. Importantly, compression due to a soft tissue mass may be missed on CT scans without contrast injection [15]. Whole-body (WB)-CT provides high-resolution images of cortical and trabecular bone with a fast scanning time and it is able to detect small (< 5 mm) lytic bone lesions [17–21]. Clinical studies addressing the use of WB-CT in comparison with other imaging techniques for SP are currently lacking. Low-dose WB-CT is currently proposed as the initial imaging technique of choice to detect bone disease in patients with MM [22].

#### Magnetic resonance imaging (MRI)

Although the sensitivity of MRI to detect lytic bone lesions is lower than that of CT, MRI is able to detect soft tissue and BM lesions and is the gold standard to detect spinal cord compression. SBP appears as an infiltration with a low T1 and a high T2 signal intensity. Moulopoulos et al. prospectively studied the role of MRI in the staging of 12 patients with SBP [23]. In order to identify other regions of BM involvement in addition to the primary SBP lesion, patients underwent spinal MRI. Additional foci of marrow replacement were found in one third of patients, and these patients showed persistent elevated serum monoclonal protein levels after radiotherapy. Conversely, patients without additional BM lesions displayed a significant reduction or disappearance of serum monoclonal protein after radiotherapy [23]. Liebross et al. confirmed these results in a second study in which 57 patients with SBP were staged with either conventional radiography alone or in combination with MRI prior to radiotherapy [24]. Among 23 patients with thoracolumbar spine disease, 7 of 8 patients, who had a solitary lesion by plain radiographs alone, developed MM in comparison with 1 of 7 patients who also had only one lesion by MRI. Together, these prospective studies underline the importance of precise staging. Consequently, excluding additional lesions is mandatory for the diagnosis of SP as per IMWG criteria [25]. The use of diffusion-weighted MRI, a new highly sensitive technique to detect and monitor tumor lesions in the BM and soft tissues, has not been reported in the management of SP.

#### Positron emission tomography/computed tomography (PET/CT)

While 18F-FDG (fluorine-18-fludeoxyglucose) PET/CT is extensively studied in MM, only a few small-scale studies have addressed its role in SP [26]. 18F-FDG PET or 18F-FDG PET/CT may show additional lesions and have therapeutic implications in 33–55% of patients with presumed SBP as patients with a normal PET/CT did not develop MM [27–29]. Salaun et al. reported that 18F-FDG PET/CT is superior to MRI for the diagnosis and follow-up of plasmacytoma patients, although it should be noted that this study was performed in the context of extramedullary spread during MM [30]. At diagnosis, the sensitivity and specificity of PET/CT was higher than that of MRI of the spine and pelvis, because PET/CT was able to detect plasmacytoma lesions with a larger scope compared to MRI, i.e., in soft tissues, skull, ribs, and limbs. Also, the specificity of PET/CT was 99% to evaluate treatment responses, compared to 89% for MRI. A second study evaluated PET/CT and axial MRI at diagnosis and follow-up in 43 patients with either EMP (10 patients) or SBP (33 patients) [26]. PET/CT at diagnosis identified 10 patients with at least two hypermetabolic lesions. Six of these patients progressed to MM, confirming a diagnostic role for PET/CT in SP. FDG-uptake was recently found to be correlated to the tumor size [31]: FDG-avid lesions are generally larger (41.4 mm) vs. FDG-negative lesions (23.5 mm) and the statistical risk of progression was higher in these FDG-avid lesions [31]. Based on these studies, the IMWG recommended PET/CT as part of the initial evaluation of patients with SP [32].

**Recommendations:** *In addition to the skeletal survey or CT, MRI or PET/CT are needed to exclude the presence of additional lesions and the use of at least one of these examinations is mandatory (Grade 1A). Both are recommended based on small prospective studies, while PET/CT has additional prognostic value. Using both PET/CT and MRI may offer complementary information. When both PET/CT and MRI are not available, a WB-CT approach can be used to exclude other lesions. (Grade 2C). To detect additional soft tissue lesions, PET/CT is recommended for patients with EMP (Grade 1B).*

## Prognostic factors

Progression of SP is defined as local recurrence, appearance of additional plasmacytoma without signs of MM, or a development of an overt MM. Different prognostic factors have been identified for SP and this has led to the proposal of different prognostic scoring methods (Table 2). However, none of these have been thoroughly validated and widely adopted.

**Table 2:** Prognostic factors

	Risk factor	Progression free survival	Reference
At diagnosis	SBP vs. EMP	Higher risk for progression to MM for SBP patients (progression rate of 65–84% at 10 years) compared to EMP patients (25–35% at 10 years)	[36, 47]
	Bone marrow plasmacytosis detected by flow cytometry*	Minimal bone marrow plasmacytosis is associated with decreased progression-free survival	[37]
	Bone marrow plasmacytosis detected by flow cytometry	About 70% of patients with minimal bone marrow plasmacytosis progress to MM with a median time to progression of 26 months	[10, 11]
	Serum SFLC ratio	An abnormal serum FLC ratio is associated with a higher risk of progression to MM compared to a normal SFLC ratio (44 vs. 26% at 5 years and 51 vs. 32% at 10 years)	[38]
	Histological abnormalities	Histologic score and degree of angiogenesis have prognostic value	[40, 41]
At follow-up	Persistence of a serum monoclonal protein after treatment	9% of progression for patients with resolved serum monoclonal protein vs. 71% of progression for patients with persistent serum monoclonal protein.	[42]

*Only studied in the context of SBP

Abbreviations: *SBP* Solitary bone plasmacytoma, *EMP* Extrameddulary plasmacytoma, *FLC* Free light chain ration, *MM* multiple myeloma

### Prognostic factors at diagnosis

EMP is usually indolent, in contrast to extramedullary spread of MM which is associated with poor prognosis [6, 7]. When compared to EMP, SBP has a worse prognosis with increased progression rates to MM, although the differences not always translate into a significant difference in overall survival (OS) [3, 4, 33–36] (Table 3). At diagnosis, the presence of minimal BM infiltration by clonal PCs is a strong prognostic factor. In a retrospective study, Warsame et al. found minimal BM infiltration, assessed by immunofluorescence, in 24 out of 61 patients and this was associated with an inferior progression-free survival (PFS 15 vs. 42 months for patients without BM involvement) [37]. These results were confirmed in recent studies in which flow cytometry was used to identify monoclonal PCs in BM samples [10, 11]. Paiva et al. detected BM clonal PCs in 49% of SBP and 38% of EMP patients. Seventy-one percent of SBP patients with minimal BM involvement progressed to MM with a median time to progression of 26 months, compared to 8% for patients without BM involvement. Of note, no significant differences were observed among EMP cases, suggesting that BM involvement has prognostic value specifically in SBP [11]. Similar progression rates (72 vs. 12.5%) and time to progression (26 months vs. not reached) were reported by Hill et al., who only evaluated patients with SBP [10].

**Table 3:** Reported outcomes for patients presenting with solitary plasmacytoma

Author		*N*	Local recurrence, %	Overall survival, % (years)	Progression-free survival, % (years)
Nahi et al. [4]	Total	191	NR	53% (8 years)	75% (2 years)
	SBP	124	NR	56% (8 years)	65% (2 years)
	EMP	67	NR	51% (8 years)	94% (2 years)
De Waal et al. [36]	Total	100	0%	68% (10 years)	50% (15 years)
	SBP	66	0%	64%(10 years)	30% (15 years)
	EMP	34	0%	77% (10 years)	88% (15 years)
Katodritou et al. [61]	Total	97	NR	78% (10 years)	59% (10 years)
	SBP	65	NR	69% (10 years)	40% (10 years)
	EMP	32	NR	86% (10 years)	50% (10 years)
Reed et al. [48]	Total	84	8%	78% (5 years)	47% (5 years)
	SBP	59	3%	76% (5 years)	56% (5 years)
	EMP	25	20%	85% (5 years)	30% (5 years)
Kilciksiz et al. [45]	Total	80	6%	73% (10 years)	NR
	SBP	57	6%	68% (10 years)	NR
	EMP	23	5%	89% (10 years)	NR
Ozsahin et al. [5]	Total	258	14%	55% (10 years)	35% (10 years)
	SBP	206	14%	52% (10 years)	28% (10 years)
	EMP	52	14%	72%(10 years)	74% (10 years)
Tsang et al. [47]	Total	46	17%	65% (8 years)	50% (8 years)
	SBP	32	22%	NR	36% (8 years)
	EMP	14	7%	NR	84% (8 years)
Liebross et al. [24]	SBP	57	4%	50% (10 years)	47% (10 years)
Liebross et al. [3]	EMP	22	7%	50% (10 years)	68% (10 years)

*NR* not reported, *SBP* solitary bone plasmacytoma, *EMP* extramedullary plasmacytoma

Alike other PC disorders, such as monoclonal gammopathy of undetermined significance (MGUS) and smoldering MM (SMM), the serum free light chain (SFLC) ratio is an independent prognostic factor in SP for progression to MM when it is outside the normal range (< 0.26 or > 1.65) [38, 39]. These results were recently confirmed by Fouquet et al., who developed a prognostic scoring method incorporating lesions on PET/CT and an abnormal SFLC ratio at diagnosis as risk factors for progression [26]. Other proposed prognostic scoring methods include histologic scoring [40] and assessment of the degree of angiogenesis [41].

### Prognostic factors during follow-up

The presence of serum monoclonal protein at the time of diagnosis has no prognostic value in SP. Conversely, persistence of the serum monoclonal protein after radiation therapy is a prognostic factor for progression to MM among SP patients with detectable serum monoclonal protein prior to treatment [23, 24, 34, 42–44]. For example, Wilder et al. reported a 10-year PFS of 29% in patients with SBP whose serum monoclonal protein did not disappear after radiotherapy, compared to 91% in patients whose serum monoclonal protein did resolve [42]. Interestingly, not only the presence of serum monoclonal protein but also its levels yield prognostic information in SBP. Dingli et al. proposed a prognostic scoring method incorporating an abnormal SFLC ratio and a persistent serum monoclonal protein level > 0.5 g/dl as adverse prognostic factors. Using this method, three groups with a different risk of progression could be identified [44]. Of note, only the persistence of serum monoclonal protein 1 year after treatment has a clear prognostic value, as its level can remain stable for several months before declining [44].

**Recommendations:** *The rate of progression to MM is higher in patients with SBP compared to patients with EMP. At diagnosis, the presence of minimal BM infiltration by aberrant plasma cells, the presence of additional hypermetabolic lesions on PET/CT, and an abnormal SFLC ratio give prognostic information and should be included in the initial assessment (Grade 1A). Persistence of a serum monoclonal protein (determined by serum electrophoresis and immunofixation) after initial radiation therapy predict a higher risk of progression to MM. Therefore, it is mandatory to monitor serum electrophoresis and immunofixation after treatment completion (Grade 1A).*

## Treatment

### Radiation therapy

Despite their locally destructive and aggressive characteristics, clonal PCs are highly sensitive to radiation and initial clinical trials confirmed the high response rates of SP to radiotherapy [24, 34, 45, 46]. Caution is warranted when analyzing older trials, because SP localization was generally only determined via conventional radiography and BM cytology. Therefore, many patients were likely understaged in these trials.

The evidence for the effectiveness of radiation therapy (Additional file 2: Table S2) is mainly based on retrospective studies as no prospective clinical trials in SP comparing radiotherapy to best supportive care or to chemotherapy have been reported. Ozsahin et al. retrospectively assessed the outcome in 206 patients with SBP and 52 patients with EMP treated with radiotherapy alone (214 patients), radiotherapy combined with chemotherapy (34 patients), or surgery alone (8 patients) [5]. The 5-year OS, disease-free survival (DFS), and local control rates were 74, 50, and 86% respectively in the entire patient population. Radiotherapy was a favorable prognostic factor for DFS and local control. The relapse rate for patients receiving a radiation dose of 40–50 Gy was 12% compared to 60% for patients who received no radiotherapy. The local response rate to radiotherapy exceeds 80–90% and appeared to be highest in tumors < 5 cm in diameter. One study reported the use of lower doses of radiotherapy and could not withhold a dose-response relationship for doses > 35 Gy, even for larger tumors [47]. However, some studies did report a relationship between the initial tumor size and local control rate. For example, Tsang et al. reported a 100% local control rate for SBP with a diameter < 5 cm compared to only 38% for SBP with a diameter > 5 cm. Whether large SBP require a higher radiation dose or combined treatment for effective local control, as these results suggest, remains to be addressed in future clinical trials. Of note, the possible relation between the administrated radiation dose and tumor size and the local control of plasmacytoma remains controversial as other retrospective studies did not confirm these findings [37, 48].

Based on the available clinical data, the recommended treatment for most patients with SP is localized, fractionated radiotherapy given at a dose of 40–50 Gy over approximately 4 weeks [49]. Therapy is generally given daily at a rate of 1.8–2.0 Gy per fraction. The treatment field should include all the involved tissues identified by imaging as well as a margin of healthy tissue (at least 2 cm). This margin is needed because the risk of tumor extension outside the initial radiation portal can enhance disease recurrence. In the case of SBP affecting vertebrae, the margin should include at least one uninvolved vertebra on either side.

### Surgery

In many instances, patients have had surgery, with complete or partial tumor removal, as part of the diagnostic procedure. Apart from the diagnostic approach, the indication of surgery are fixation of fractures, decompressive laminectomy, or spine stabilization. The introduction of modern spinal fixation and stabilization methods, such as vertebroplasty and kyphoplasty, allows for a surgical solution for patients who develop vertebral fractures, vertebral instability, neurological complications, or a combination of these. Radiotherapy can be delayed until after surgery but it is still required because tumor excision without subsequent radiotherapy results in a very high rate of local recurrence [46].

### Chemotherapy

The role of adjuvant chemotherapy after radiation therapy in the treatment of SP remains controversial precluding a definite recommendation. In a study on 32 SP patients, Holland et al. observed that adjuvant chemotherapy did not affect the incidence of progression to MM (53% for SBP and 36% for EMP) but did delay progression from 29 to 59 months [43]. Similarly, Aviles et al. reported improved outcome in a small randomized prospective clinical trial on SBP when patients received adjuvant melphalan and prednisolone for 3 years [50]. After a median follow-up of 8.9 years, OS was 88% for patients receiving adjuvant chemotherapy, compared to 46% in the control group. Of note, BM infiltration in these patients was assessed by morphology alone, which does not have the sensitivity required to detect minimal infiltration. Conversely, other studies indicate that adjuvant chemotherapy for SP has no benefit [51, 52]. Also, autologous stem cell transplantation has been evaluated in high-risk SP patients but, although the initial results were promising, the small number of patients in this study precludes drawing definitive conclusions [53]. Some of the authors of these guidelines propose the use of adjuvant chemotherapy for bulky SP (> 5 cm), but this approach is not supported by previous studies. Similar to other plasma cell precursor entities (MGUS and SMM), bisphosphonates are recommended for those patients with confirmed osteoporosis on dual-energy X-ray absorptiometry scan in doses used for patients with osteoporosis [54]. Systemic use of bisphosphonates has not been studied in the context of SP but does not delay tumor progression in the SMM setting [55]. They can be considered for patients with a threatening fracture due to a SBP.

**Recommendations:** *Radiation therapy is the standard treatment for SBP and EMP (Grade 1A). A total fractionated dose of 40–50 Gy should be given, and a margin of at least 2 cm should be employed (Grade 1A). For patients with SBP, surgery should be reserved for the treatment of pathological fractures, neurological complications, or lesions with a high chance for fracture or instability. For patients with EMP, surgery might be able to resect large and well-defined masses but should be followed by radiotherapy. Due to the limited available data, adjuvant chemotherapy is not recommended for patients with SP. Adjuvant chemotherapy can be considered for patients with persistent disease, based on PET/CT, after initial radiotherapy (Grade 2C).*

## Response assessment

There are currently no guidelines for the assessment of treatment responses in SP. We suggest that the definitions that are currently applied in MM can be used in the setting of SBP, while RECIST criteria can be used for assessing treatment responses in EMP [56, 57] (Table 4). For patients who have detectable serum monoclonal protein at diagnosis, the response can be assessed similar to the uniform IMWG response criteria for MM [58]. Serum and urine electrophoresis and immunofixation as well as serum free light chain analysis should be regularly performed during follow-up. As discussed, serum monoclonal protein can remain stable for several months before declining. In case of increasing serum monoclonal protein levels or the appearance of myeloma-associated end-organ damage, a new bone marrow sample should be taken in order to quantify the PC infiltration and to detect genetic abnormalities. The sensitivity of PET/CT for the evaluation of treatment responses is higher than that of MRI. A persistent high uptake on 18F-FDG PET/CT indicates the presence of residual disease. Low tracer uptake at irradiated sites can be due to bone remodeling and is not always associated with a poor prognosis. Preferentially, the same imaging technique should be used for diagnosis and for the evaluation of treatment responses.

**Table 4:** Definition of treatment responses

Complete response (CR)	Complete disappearance of all previously observed abnormalities on radiographic imaging. For patients with a secretory plasmacytoma, a disappearance of monoclonal protein from serum and/or urine. For SBP, the initial radiological abnormalities on MRI or CT should regress or stabilize during an observation time of at least 12 months to fulfill the requirements for a CR. For EMP, the disappearance of soft tissue mass is required for the definition of CR
Very good partial response (VGPR)	A CR with regard to clinical and radiological signs, but with a positive immunofixation or ≥ 90% reduction in serum monoclonal protein plus urine monoclonal protein level < 100 mg/24 h.
Partial response (PR)	A ≥ 50% decrease in serum and/or urine monoclonal protein. For non-secretory SP, radiological features (MRI/CT) or local assessment is needed. In EMP patients, a 30% decrease in the diameter of target lesions should be observed.
Stable disease (SD)	Insufficient shrinkage to qualify for PR nor sufficient increase to qualify for PD.
Progressive disease (PD)	The development of new lesions or an increase of at least 20% in the size of existing lesions, the apparition of a myeloma defining event, and finally an increase of > 25% from lowest response value in serum and/or urine monoclonal protein.

**Recommendations:** *Treatment response should be assessed according to the IMWG criteria for patients with measurable serum/urine parameters (Grade 1B), although serum monoclonal protein can remain stable for several months. For soft tissue lesions, these criteria should be combined with the RECIST criteria (Grade 2C). During further follow-up of the disease, the same imaging (preferentially a whole body technique) method should be used, but one should be aware that the sensitivity of PET/CT is higher than that of MRI.*

## Future direction for clinical research

The current guidelines in SP are based on small-scale studies because prospective clinical trials are hampered by the low incidence of SP. Still, larger studies are needed to further establish and refine the current diagnosis and treatment guidelines. Large prospective clinical trials to evaluate addition of systemic treatment (including novel agents) to radiation therapy are needed to define the optimal treatment approach of patients presenting poor prognostic factors such as tumor size and the presence of clonal BMPCs. Moreover, different imaging techniques should be compared for SP diagnosis and follow-up. While low-dose WB-CT is used in MM, studies investigating this technique in SP are still lacking. At this time, PET/CT is considered the imaging technique of choice, but the introduction of diffuse weighted MRI could improve the ability of MRI to discriminate between active and inactive lesions. A multi-center SWOG trial (ClinicalTrials.gov: NCT00109889) comparing PET/CT with MRI in SP was closed in 2007 but its results are still unknown. In 2017, there is no scientific evidence that patients with an initial BM infiltration require additional therapy, although they are at high risk of progression, particularly those with aberrant phenotype. Accordingly, in smoldering MM (SMM) a high-risk group of patients with > 70% risk of progression to MM in 2 years could be identified by immunophenotyping of aberrant PCs. It has been shown that early treatment prevents progression to MM in these high-risk SMM patients [59]. Mateos et al. demonstrated not only a superior PFS, but also OS in patients treated with lenalidomide-dexamethasone, as compared to observation [60].

Although it is currently unknown, whether such a paradigm is applicable to SBP, the rate of progression to MM in SBP patients with occult BM infiltration is comparable to that observed in high-risk SMM. Therefore, a randomized trial with similar design comparing treatment for a limited period vs. observation seems justified. A regular follow-up by blood and urine testing is required for these patients and a new BM aspiration and biopsy should be performed when a progression is suspected.

Advanced and sensitive techniques for the detection of monoclonal PCs are being developed and are already used in daily practice to detect MRD in the follow-up of MM. However, prospective trials with these new techniques in the diagnosis and follow-up of SP are lacking. Multiparametric flow cytometry uses a standard set of monoclonal PC-associated markers, is quantitative, includes an internal quality control of the sample cellularity and hemodilution, and is applicable for virtually every patient. Also, PCR-based techniques such as quantitative allele-specific oligonucleotide PCR (ASO-qPCR) and next-generation sequencing of VDJ sequences remain unexplored in SP.

While FISH studies have confirmed that SP is part of the spectrum of plasma cell disorders, they could not identify prognostic subgroups as in MM. Future comparisons between monoclonal plasma cells obtained from SP and BM (if a minimal infiltration is present) would allow identification of additional, secondary genetic aberrations that drive tumor progression. More advanced techniques such as single nucleotide polymorphism (SNP) or gene expression arrays will also contribute to a better understanding of the tumor biology. Inclusion of more patients in these studies will facilitate prognostic stratification and identification of patients with a high risk of progression that could benefit from a systemic treatment to postpone disease progression.

As for prognostic scoring methods, only minimal BM aberrant plasmacytosis has been extensively validated as a prognostic factor in SBP for progression to MM. The identification and validation of additional risk factors, such as those used in SMM, is of interest to better identify those patients that might benefit from adjuvant therapy or to delay progression to MM.

Finally, additional clinical trials to address the role of adjuvant chemotherapy for SP are needed. Of note, the treatment approaches in all reported studies included conventional chemotherapy. Studies with novel potent MM agents, such as proteasome inhibitors (bortezomib, ixazomib, and carfilzomib), immunomodulatory agents (thalidomide, lenalidomide, and pomalidomide) or monoclonal antibodies (elotuzumab and daratumumab) are currently lacking. Focus on those patients who are at high risk for progression to MM (i.e., the detection of BM clonal PC cells by flow cytometry, more than one hypermetabolic lesion at PET/CT plus abnormal FLC ratio, residual paraprotein after radiation therapy, and bulky tumor mass > 5 cm) is warranted.

## Conclusion

Solitary plasmacytoma belongs to a spectrum of plasma cell disorders and is defined by a single mass of monoclonal plasma cells that can be found in the bones or in the extramedullary regions. We updated the recommendations on the diagnosis and management and proposed a new definition of response assessment. While radiotherapy remains the recommended treatment, several risk factors for early progression to multiple myeloma have been identified. Further studies evaluating adjuvant treatment regimens (based on novel agents) are urgently needed for these high-risk patients.

## Additional files

Additional file 1: Table S1. Grading of recommendations. (DOCX 12 kb)

Additional file 2: Table S2. Results of radiotherapy in patients presenting with SP [62, 63]. (DOCX 36 kb)

## Abbreviations

18F-FDG: Fluorine-18-fludeoxyglucose
ASH: American Society for Hematology
ASO-qPCR: Quantitative allele-specific oligonucleotide PCR
BM: Bone marrow
CR: Complete response
CT: Computed tomography
EMN: European Myeloma Network
EMP: Extramedullary plasmacytoma
GRADE: Grading of Recommendations Assessment, Development and Evaluation
IMWG: International Myeloma Working Group
MGUS: Monoclonal gammopathy of undetermined significance
MM: Multiple myeloma
MRI: Magnetic resonance imaging
PCR: Polymerase chain reaction
PD: Progressive disease
PET/CT: Positron emission tomography–computed tomography
PR: Partial response
RECIST: Response Evaluation Criteria In Solid Tumors
SBP: Solitary bone plasmacytoma
SD: Stable disease
SFLC: Serum-free light chain
SP: Solitary plasmacytoma
VGPR: Very good partial response
WB-CT: Whole body computed tomography
