# Supplementary material for: Diagnosis, treatment, and response assessment in solitary plasmacytoma: updated recommendations from a European Expert Panel
Source: J Hematol Oncol. 2018 Jan 16;11:10. doi: 10.1186/s13045-017-0549-1 (PMC5771205; doi:10.1186/s13045-017-0549-1)
Supplement: Supplementary file 1 — Grading of recommendations. (DOCX 12 kb) [file 13045_2017_549_MOESM1_ESM.docx]

**Table S1: Grading of recommendations**

| **Strength of recommendations** | **Quality of evidence** |
| --- | --- |
| ***Strong (Grade 1):*** Strong recommendations are made when there is confidence that the benefits of an intervention outweigh the potential risks. Grade 1 recommendations can be applied uniformly to most patients. Regarded as ‘recommended’. | ***(A) High:*** Further research is very unlikely to change confidence in the estimate of effect. Current evidence derived from randomized clinical trials or high quality observational studies. |
| ***Weak (Grade 2):*** When the risks and benefits are less certain, a weak recommendation is made. Grade 2 recommendations require judicious application to individual patients. Regarded as ‘suggested’. | ***(B) Moderate:*** Further research may well have an important impact on confidence in the estimate of effect and may change the estimate. Current evidence derived from randomized clinical trials with important methodological flaws. |
|  | ***(C) Low:*** Further research is likely to have an important impact on confidence in the estimate of effect and is likely to change the estimate. Current evidence from observational studies, case series or just opinion. |
