# Supplementary material for: Diagnosis, treatment, and response assessment in solitary plasmacytoma: updated recommendations from a European Expert Panel
Source: J Hematol Oncol. 2018 Jan 16;11:10. doi: 10.1186/s13045-017-0549-1 (PMC5771205; doi:10.1186/s13045-017-0549-1)
Supplement: Supplementary file 2 — Results of radiotherapy in patients presenting with SP [62, 63]. (DOCX 36 kb) [file 13045_2017_549_MOESM2_ESM.docx]

**Table S2: Results of radiotherapy in patients presenting with SP**

| **Author** | **Diagnosis** | **N** | **Radiation (Gy)** | **Local progression** | **Disease-free**  **survival *%*** | **OS, %** | **PFS, %** |
| --- | --- | --- | --- | --- | --- | --- | --- |
| Katodritou *et al.,* 2014 [[61](#_ENREF_61)] | SBP | 65 | 40.0 (24–55) | NR | 40% (10 years) | 69% (10 years) | 50% (10 years) |
| Warsame *et al.,* 2012 [[37](#_ENREF_37)] | SBP | 91 | 46.0 (20–60) | 7.8% | 78% (5 years) | 45% (5 years) | NR |
| S. Kilciksiz *et al.,* 2008 [[45](#_ENREF_45)] | SBP | 57 | 46.0 ( 32–59) | 6% | NR | 68% (10 years) | NR |
| Tsang *et al.,* 2001 [[47](#_ENREF_47)] | SBP | 32 | 35.0 (30–50) | 22% | 44% (8 years) | NR | 50% (8 years) |
| Reed *et al.,* 2011 [[48](#_ENREF_48)] | SBP | 59 | 45.0 (36–54) | 3% | NR | 76% (5 years) | 56% (5years) |
| Wilder *et al.,* 2002 [[42](#_ENREF_42)] | SBP | 60 | 46.0 (30-70) | 6.3% | NR | 59 % (10 years) | 38% (10 years) |
| Frassica *et al.,* 1989 [[62](#_ENREF_62)] | SBP | 46 | 39.75 (20–70) | 11% | 25 % (10 years) | 45% (10 years) | NR |
| Knobel *et al.,* 2006 [[46](#_ENREF_46)] | SBP | 201 | 40.0 (20–64) | 13% | 25% (10 years) | 50% (10 years) | 28% ((10 years) |
| Liebross *et al.,* 1998 [[24](#_ENREF_24)] | SBP | 57 | 50.0 (30–70) | 4% | NR | 50% (10 years) | 47% (10y years) |
| Katodritou *et al.,* 2014 [[61](#_ENREF_61)] | EMP | 32 | 40.0 (24–55) | NR | 50% (10 years) | 89% (10 years) | 70% (10 years) |
| Kilciksiz *et al.,* 2008 [[45](#_ENREF_45)] | EMP | 23 | 46.0 ( 32–59) | 6% | NR | 89% (10 years) | NR |
| Reed *et al.,* 2011 [[48](#_ENREF_48)] | EMP | 25 | 45.0 (36–54) | 20% | NR | 85% (5 years) | 30% (5 years) |
| Galieni *et al.,* 2000 [[34](#_ENREF_34)] | EMP | 46 | 46.0 (30-60) | 7.5% | 83% (15 years) | 78% (15 years) | 85% (5 years) |
| Ozsahin *et al.,* 2006 [[5](#_ENREF_5)] | EMP | 52 | 40 (20-66) | 14% | 55% (10 years) | 72%(10 years) | 74% (10 years) |
| Bachar *et al.,* 2008 [[63](#_ENREF_63)] | EMP | 68 | 35 (10-50) | 19% | 41% (10 years) | 56% (10 years) | 72% (10 years) |
